# Incorporating validated alcohol and drug screening instruments in the electronic health record

**DOI:** 10.1186/1940-0640-10-S2-O47

**Published:** 2015-09-24

**Authors:** George Allen Tindol, Krista Gonzales, Kasra Sedarati, Cara Smith

**Affiliations:** 1 Department of Internal Medicine Education, Memorial University Medical Center/Mercer University School of Medicine (Savannah Campus), 1101 Lexington Avenue, Savannah, Georgia, 31404, USA

## Background

We sought to implement validated alcohol and drug use screening tools into the Electronic Health Record (EHR) without the aid of specialized information technology (IT) support.

## Material and methods

Since October of 2012, when our outpatient clinic transitioned to an EHR, we implemented the AUDIT and DAST-10 questionnaires as Epic® SmartPhrases.

A high score on either questionnaire prompts a "brief intervention" (BI), which includes offering the patient a printed brochure. To our SmartPhrases, we added hyperlinks (to http://www.sbirtonline.org and http://www.drugabuse.gov) to make pertinent brochures immediately available.

To "charge capture" and retrieve BIs, we used the Current Procedural Terminology (CPT) codes for alcohol and drug counseling.

## Results

Of 155 randomly selected patient encounters prior to using the EHR, there were no encounters with a validated alcohol use screening instrument, and in only 1 encounter (0.6 %) was there a valid BI. After transitioning to the EHR and educating residents, 43 of 97 (44.3%, p < 0.05) randomly selected patient encounters included a validated alcohol use screening instrument, but only 4 of 97 (4.1%) contained evidence of a BI. Over time, with continued training, including conferences and reminders from attending physician "champions," residents performed more BIs. For the year April 2014 through March 2015, documented BIs averaged 11 per month (1.3% of total monthly patient encounters).

## Conclusions

Though the resulting workaround process may not be "seamless," EHR users can independently implement validated screening questionnaires. With proper IT expertise, Best Practice Advisory/Health Maintenance Reminder (BPA/HMR) "pop-ups" can increase the automaticity of screening, enforce the necessity of screening, and minimize the chances of "losing" documentation of screening (such as when residents fail to "charge capture.") The BPA/HMR method, with automatic periodic reminders which the provider must address - in perpetuity - before moving to the next step in encounter documentation, should aid sustainability of alcohol and drug use screening and intervention programs.

